# Transcriptomic landscape of Atlantic salmon (*Salmo salar* L.) skin

**DOI:** 10.1093/g3journal/jkad215

**Published:** 2023-09-19

**Authors:** Lene R Sveen, Nicholas Robinson, Aleksei Krasnov, Rose Ruiz Daniels, Marianne Vaadal, Christian Karlsen, Elisabeth Ytteborg, Diego Robledo, Sarah Salisbury, Binyam Dagnachew, Carlo C Lazado, Torstein Tengs

**Affiliations:** Nofima, Fish Health, Tromsø NO-9291, Norway; Nofima, Fish Health, Tromsø NO-9291, Norway; School of BioSciences, The University of Melbourne, Melbourne 3010, Australia; Nofima, Fish Health, Tromsø NO-9291, Norway; The Roslin Institute and Royal (Dick) School of Veterinary Studies, The University of Edinburgh, Edinburgh EH25 9RG, UK; Nofima, Fish Health, Tromsø NO-9291, Norway; Nofima, Fish Health, Tromsø NO-9291, Norway; Nofima, Fish Health, Tromsø NO-9291, Norway; The Roslin Institute and Royal (Dick) School of Veterinary Studies, The University of Edinburgh, Edinburgh EH25 9RG, UK; The Roslin Institute and Royal (Dick) School of Veterinary Studies, The University of Edinburgh, Edinburgh EH25 9RG, UK; Nofima, Fish Health, Tromsø NO-9291, Norway; Nofima, Fish Health, Tromsø NO-9291, Norway; Nofima, Fish Health, Tromsø NO-9291, Norway

**Keywords:** spatial transcriptomics, fish skin, RNAseq, histology, gene expression, epithelium, connective tissue, fin, bone, mesenchyme

## Abstract

In this study, we present the first spatial transcriptomic atlas of Atlantic salmon skin using the Visium Spatial Gene Expression protocol. We utilized frozen skin tissue from 4 distinct sites, namely the operculum, pectoral and caudal fins, and scaly skin at the flank of the fish close to the lateral line, obtained from 2 Atlantic salmon (150 g). High-quality frozen tissue sections were obtained by embedding tissue in optimal cutting temperature media prior to freezing and sectioning. Further, we generated libraries and spatial transcriptomic maps, achieving a minimum of 80 million reads per sample with mapping efficiencies ranging from 79.3 to 89.4%. Our analysis revealed the detection of over 80,000 transcripts and nearly 30,000 genes in each sample. Among the tissue types observed in the skin, the epithelial tissues exhibited the highest number of transcripts (unique molecular identifier counts), followed by muscle tissue, loose and fibrous connective tissue, and bone. Notably, the widest nodes in the transcriptome network were shared among the epithelial clusters, while dermal tissues showed less consistency, which is likely attributable to the presence of multiple cell types at different body locations. Additionally, we identified *collagen type 1* as the most prominent gene family in the skin, while *keratins* were found to be abundant in the epithelial tissue. Furthermore, we successfully identified gene markers specific to epithelial tissue, bone, and mesenchyme. To validate their expression patterns, we conducted a meta-analysis of the microarray database, which confirmed high expression levels of these markers in mucosal organs, skin, gills, and the olfactory rosette.

## Introduction

Atlantic salmon (*Salmo salar* L.) is one of the most important farmed fish species worldwide. With its production of 2.7 million tons in 2020, Atlantic salmon accounted for 32.6% of marine and coastal aquaculture of all finfish species (FAO 2022). It is a cold-water species that is native to the North Atlantic Ocean and its adjacent seas. Norwegian salmon farming industry has been facing persistent challenges associated with skin pathogens and ulceration (Sveen *et al*. 2018; Sommerset *et al*. 2022), where host responses leading to pathogen clearance and tissue repair are crucial for the restoration of skin barrier function (Sveen *et al*. 2020). These skin health–related challenges present a significant welfare issue that must be addressed through a better understanding of the immunology and physiology of salmon skin.

The skin is the outer layer of the body (Hawkes 1974; Elliott 2011), which separates and protects the animal from its environment. In fish, the skin is continuous with the lining of all the body openings, including the head and the fins. Further, the skin has similarities but also differences depending on body position; however, in general, 2 tissue types dominate: epithelial tissue (epidermis) and connective tissue (dermis).

In Atlantic salmon, the epidermis primarily contains epithelial cells and mucous-secreting cells (Sveen *et al*. 2021b), which serve as a barrier toward the external environment (Whitear 1986a; Doyle *et al*. 2022). The barrier function of the epithelial tissue is both external and internal. The external barrier is maintained through production and secretion of a protective mucus layer. The mucus layers contain a variety of antimicrobial peptides, proteases, and lipids protecting against numerous disease-causing agents, such as bacteria, parasites, and viruses (Esteban 2012). Intercellular protection is achieved through a network of tight junction proteins, which are critical to separate tissue spaces and regulate movement of solutes across the epithelium (Doyle *et al*. 2022). In addition, teleost fish possess an adaptive immune system associated with each of their mucosal body surfaces, in the skin referred to as skin-associated lymphoid tissue (SALT) (Salinas 2015). Small populations of B and T cells are present both in epithelial (Xu *et al*. 2013) and dermal tissue (Karlsen *et al*. 2023), depending on the state of the organ.

The dermis provides mechanical support, flexibility, and resilience to the integument (Whitear 1986a). The dermis has a different organization at different body sites, which is important for body functions. At the main body, the overlapping scales provide physical protection and improve locomotion (Oeffner and Lauder 2012; Wainwright and Lauder 2017). The scales rest in pockets of loose connective tissue, which is well vascularized and rich in fibroblasts, melanophores, chromatophores, nerve cells, sensory cells, and immune cells (Le Guellec *et al*. 2004). The loose connective tissue is anchored in the dense connective tissue. The dense connective tissue is primarily a structural tissue, where the arrangement of the collagen fibers is particularly important for flexibility and locomotion, where muscular contraction produces tendon-like responses in the skin (Szewciw and Barthelat 2017). The dense connective tissue rests on a layer of adipose tissue (hypodermis). The dermal tissue of the fins and at the head lacks scales, adipose tissue, and dense connective tissue; instead, bony features and loose connective tissue provide most of the tissue support (Smith *et al*. 1994; Puri *et al*. 2018; König *et al*. 2019). In addition, the fins stands out with its mesenchyme, a type of embryonic connective tissue that gives rise to a variety of cell types, including fibroblasts, chondrocytes, and osteoblasts, and is one of the reasons why fins may regenerate after amputation (Pfefferli and Jaźwińska 2015).

In recent years, significant progress has been made in understanding the molecules and underlying processes in Atlantic salmon skin (Micallef *et al*. 2012). However, most studies have focused on investigating the molecular repertoire of the skin, without considering its spatial expression patterns. Spatial expression studies have been limited to a few numbers of targets, primarily with immune histochemistry techniques (Holm *et al*. 2017; Sveen *et al*. 2019). However, available antibodies which work well in salmon skin are scarce. As a result, our understanding of the precise spatial organization and differential responses of specific tissue types within the skin has remained limited.

In our previous research, we took a step further by employing a more refined approach that involved the separation of epithelial and dermal tissues prior to transcriptome analysis (Sveen *et al*. 2021a; Karlsen *et al*. 2023). This enabled us to uncover marked differences in the responses of these distinct tissue types to parasite and bacterial infections. The findings strongly suggest that different tissue components within the skin possess unique and specialized response mechanisms when faced with various stimuli.

By expanding our understanding of the spatial expression patterns and functional diversity within the skin, we can gain deeper insights into the complex interplay between different tissue types and their specific roles in maintaining skin health and defense mechanisms. Spatial transcriptomics is an innovative technology that combines traditional transcriptomics with spatial information. It is described as a spatially resolved, high-dimensional assessment of gene transcription, where the gene transcripts are spatially localized and quantified in their original position within the tissue (Williams *et al*. 2022). Commercialized techniques such as Visium Spatial Gene Expression released by 10x Genomics (Ståhl *et al*. 2016), as well as GeoMx (Merritt *et al*. 2020) and CosMx (He *et al*. 2021) by NanoString, have now made spatial transcriptomics more accessible, though still remains costly.

The Visium technique operates by pulling down poly-A mRNA onto a grid of barcoded spots, ultimately covering the transcriptome of a sample. If the samples comprise mRNA from various eukaryotic species (prokaryotes lack mRNA poly-A tail), the opportunity emerges to simultaneously conduct spatial transcriptomic analysis for multiple species within a single sample. This scenario holds potential for instances like examining salmon lice-infected skin (Robinson *et al*. 2022), amoebic gill disease, and proliferative kidney disease.

The NanoString technologies offer higher resolution than the Visium platform but currently rely on a targeted approach, with a limited subset of custom-made barcoded probes for nonmodel species. Depending on the probe design, NanoString may also capture transcriptomic patterns from multiple species concurrently, including prokaryotes. Both techniques depend on Illumina sequencing (Williams *et al*. 2022). Considering that Atlantic salmon possesses a relatively well-annotated reference genome, such as assembly Ssal_v3.1, Bioproject PRJNA788898 (Lien *et al*. 2016), untargeted transcriptomic approaches like Visium Spatial Gene Expression showcase promise as a tool for genetic investigations (Robinson *et al*. 2022).

Here, we present the high-resolution spatial transcriptomes of “naive” skin samples from 4 distinct body sites in Atlantic salmon using the 10x Genomics Visium platform. The spatially resolved transcriptomic map elucidates the molecular repertoire of the skin, emphasizing key molecules crucial for barrier functions. This comprehensive data set is a molecular toolbox that can be explored to develop interventions aimed at improving the barrier functionality of the skin against biological and environmental challenges, thereby improving fish welfare.

## Materials and methods

### Tissue sampling

**Fig. 1.**
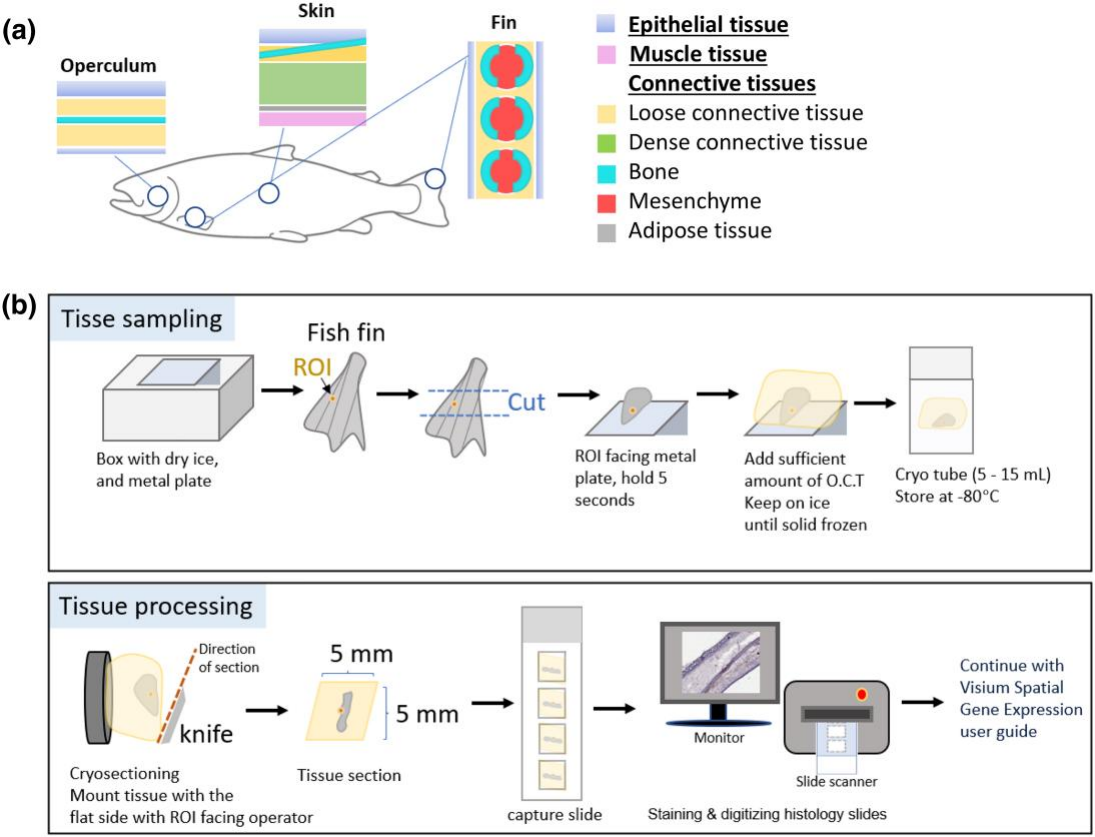
Tissue sampling and optimization. a) The 4 tissue sampling sites are marked by circles, with schematic illustrations of the main tissue types present in the operculum, skin at the lateral line, pectoral fin, and dorsal fin. b) The samples (maximum size 5 × 5 mm) were held in an upright position for 5 s on a frozen metal plate, the ROI facing the metal plate. The specimens were embedded in O.C.T. and held on dry ice until fully frozen and transferred to appropriate tubes. For processing of the samples, the O.C.T.-embedded tissue was mounted on to the cryostat sample holder with the flat surface and ROI facing the operator. Skin and fins were cut into 10-μm-thick cryo sections and mounted on expression slides. Tissues were scanned with Aperio CS2 (Leica, USA).

The sampled fish were part of the routine production at the University of Life Sciences (As, Norway). Prior to tissue collection, 2 Atlantic salmon (150 g), 1 male and 1 female, reared in freshwater, were netted from their respective fish tanks and sedated in a bucket with a low dose of Aqui-S (4 mL Aqui-S/15 L of H_2_O) (Scanvacc, Norway), until loss of equilibrium, and euthanized with a blow to the head. Tissue samples were collected from 4 distinct locations, left side of the body, including the operculum, caudal and dorsal fin, and scaly skin from the flank of the fish close to the lateral line (Fig. 1a). From here on, scaly skin is referred to only as skin. Each of the tissue samples was ∼5 mm long and <5 mm wide, so that they would fit into the 6 × 6 mm capture window on the Visium expression slides.

The Visium protocol (Visium Spatial Protocols—Tissue Preparation Guide, CG000240 RevB) was adjusted to address the difficulties encountered in making sections through the samples. This adjustment involved employing TissueTek (Sakura Finetek, USA) optimal cutting temperature (O.C.T.) compound embedding before freezing and sectioning the tissues (Fig. 1b). The purpose of this adjustment was to ensure the production of high-quality tissue sections from all body sites.

In brief, a metal plate was precooled on dry ice. Tissue samples were cut from the fish and immediately transferred to the metal plate. On the metal plate, the samples were held in an upright position for 3–5 s to ensure the proper vertical positioning of the tissue before the application of O.C.T. media. Subsequently, the metal plate with O.C.T.-embedded samples was held on dry ice until samples were fully frozen (Fig. 1b). Throughout the procedure, particular attention was given to positioning the samples so that the region of interest (ROI) faced the flat metal surface. This step ensured the creation of a uniform surface that would be easily identifiable when subsequently mounting the samples for cryosectioning. The fully embedded and frozen tissue was subsequently transferred to 15-mL Falcon tubes (Corning Life Sciences, USA) and stored at –80°C until further processing.

### Tissue optimization

Before the tissue optimization step, a quality assessment was performed on 2 samples to evaluate their RNA Integrity Number (RIN) using the 2000 Bioanalyzer from Agilent Technologies, USA. Both samples demonstrated satisfactory RIN values of 8.6 and 9.8, signifying high-quality RNA preservation.

Skin and fins were sectioned into 10-μm-thick cryo sections, longitudinal section for the skin and operculum, and cross sections for the fins. The optimization of tissue permeabilization was performed using Visium Spatial Tissue Optimization Reagents Kit according to the protocol provided (10x Genomics, Pleasanton, CA, USA). A total of 2 optimization slides were conducted, with each slide containing 8 capture frames. On the first slide, fin tissue was subjected to permeabilization times of 5, 10, 15, 20, 25 35, and 45 min. The fluorescent cDNA signal was manually assessed with a Leica CTR 6000 fluorescent microscope (Leica, USA). A good cDNA fluorescent signal was obtained from the epithelial layer within 10–25 min of permeabilization time, whereas in comparison, the fluorescent signal was weaker for the dermal tissue. The process was repeated, using permeabilization times of 20, 25, 30, and 35 min, for parallel section of 1 skin and 1 fin samples. Based on the intensity of the fluorescent signal in the epithelial and dermal tissue, a permeabilization time of 20 min was chosen for the tissue expression slides.

After selecting optimization time, 8 samples (skin, caudal fin, dorsal fin, and operculum) from 2 individuals were mounted onto 2 Visium expression slides (10x Genomics) and stored at −80°C until hematoxylin and eosin (H&E) staining. Tissue staining and library preparation were conducted according to the Visium Spatial Gene Expression User Guide (10x Genomics). Tissues were scanned with Aperio CS2 (Leica, USA).

### Sequence mapping, cell population identification, and visualization of gene expression

Libraries were sequenced on NovaSeq 6000 SP flow cell (Illumina, USA) at the Norwegian Sequencing Center as 50 bp paired end reads. Sequencing was done using the following cycles: read 1, 28 cycles, i7 Index; 10 cycles, i5 Index; 10 cycles; and read 2, 90 cycles (Visium Spatial Gene Expression User Guide; 10x Genomics). Reads were aligned to the Atlantic salmon genome (version Ssal_v3.1, INSDC Assembly GCA_905237065.2) using the software Space Ranger (version 1.3.1; 10x Genomics, USA). High-resolution JPG images from each of the associated tissue sections were aligned to the reads by default settings. Optimal number of tissue clusters and cluster membership of spots was defined using graph-based clustering [modified python implementation of the augmented implicitly restarted Lanczos bidiagonalization algorithm (IRLBA) (Baglama and Reichel 2005)] in Space Ranger. Transcripts defining tissue clusters were filtered using the following criteria: only upregulated transcripts (relative to the other clusters), adjusted *P*-value (Benjamini–Hochberg procedure) < 0.1, and mean barcoded unique molecular identifier (UMI) count > 1. Genes that showed differential expression within a population of cells were referred to as differentially expressed genes (DEGs).

For practical reasons, some figures only display examples from 1 specimen. Expression levels of genes (single genes or average gene expression of multiple genes) were visualized using 10x Genomics Loup Browser v6.0.0.

### Gene markers

Tissue clusters and DEGs were visualized in 10x Genomics Loupe Browser, and the visual overlay of gene expression with the tissue of interest was assessed by trained histologists. The spatial expressional pattern was assessed in all 8 samples before gene markers for epithelia tissue, bone, and mesenchyme were selected manually. Genes with missing annotations or showing inconsistent expression patterns between samples were excluded as gene markers.

### Search for gene markers in Nofima’s STARS database

A search for gene markers, collagens, and interfilamentous proteins was linked to our selection microarray database STARS (Krasnov *et al*. 2011). Transcriptomes were compared in 2 stages. First, normalization was performed for each tissue by calculating the overall average intensity and multiplying each point by a correction factor so that the average intensities of all arrays were equal. The ratio of intensity to the average intensity for a given gene was calculated at each point. A global normalization of the means for tissues and cell types was then performed. Mean values were calculated for genes and subtracted from each data point. Data are Log2 AVG fold change of tissue to the mean of all tissues. GeneBank and STARS annotations are given in Supplementary File 1. It is assumed that the intensity of the hybridization signal minus the background is proportional to the number of transcripts. At the time of the search, the database housed a total of 177 experiments with >3,000 arrays (44k genome-wide Salgeno platform).

## Results and discussion

### Performance of spatial transcriptomics on Atlantic salmon skin tissue sections

**Fig. 2.**
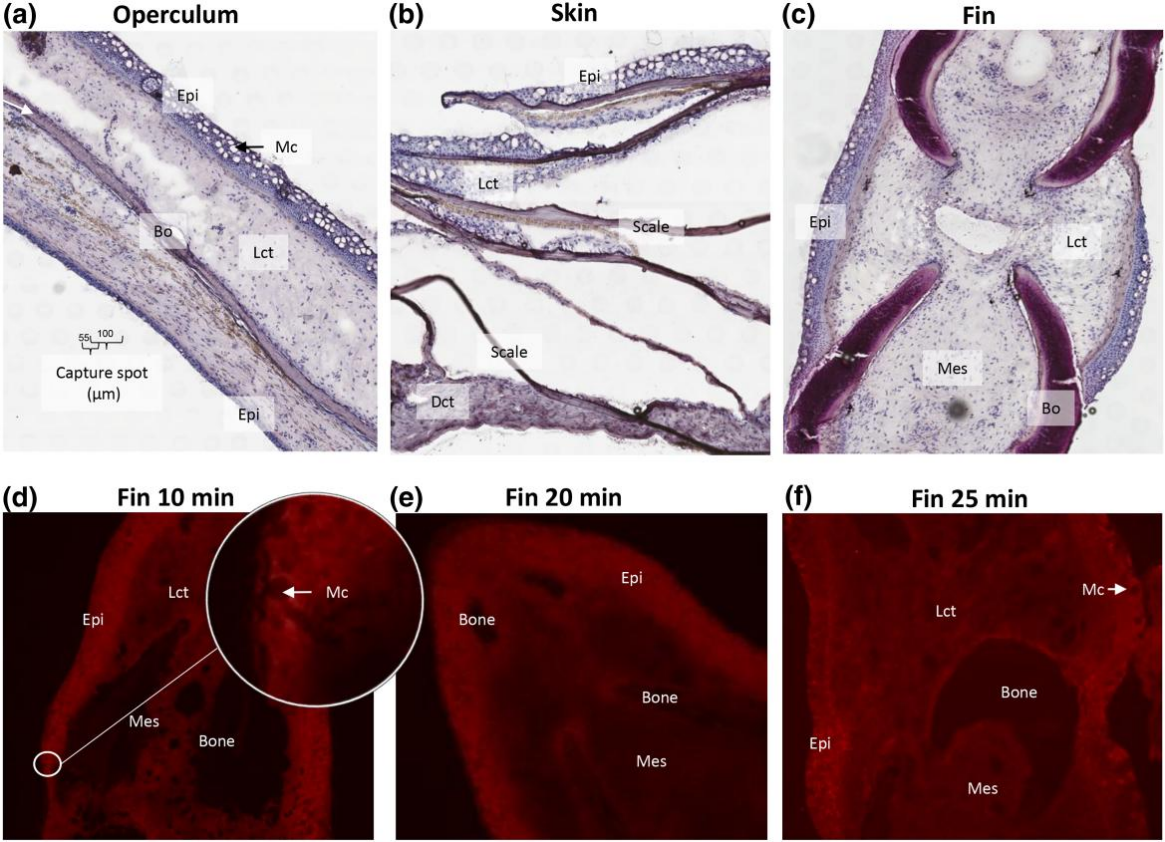
Tissue sections and permeabilization time. a–c) Frozen tissue sections, 10 µm, of operculum, skin, and fin samples were sectioned onto Visium expression slides and stained with H&E. Abbreviations: Epithelium (Epi), loose connective tissue (Lct), dense connective tissue (Dct), mucous cell (Mc), bone (Bo), and mesenchyme (Mes). Capture spot diameter and center-to-center distance are indicated in a. d) Fluorescent cDNA print of pectoral fin (10 min optimization time). Insert with higher magnification shows epithelial tissue with mature mucous cells displayed as circles with low fluorescent signal. e and f) Similar to d with 20 and 25 min permeabilization time. The intensity of the fluorescent signal indicates cDNA/mRNA yield. At all timepoints, the intensity of the fluorescent signal was higher in the epithelial layer when compared with the dermal layer.

Freezing and embedding of tissue samples prior to cryosection was an important step to maintain tissue morphology and RNA quality. We adapted the original protocol, where tissue samples were frozen in a bath of isopentane and liquid nitrogen prior to O.C.T. embedding, to direct embedding of tissue samples in O.C.T. and freezing of embedded tissue on dry ice. The O.C.T. compound stabilized the tissue during the freezing process, which was crucial for obtaining high-quality tissue sections (Fig. 2a–c). This adaptation was necessary as cryosectioning of the samples was particularly challenging, due to a combination of soft and hard tissue types.

Before conducting the Visium gene expression protocol and generating libraries, it was important to establish the optimal permeabilization time for the tissue sections. During this process, the tissue was sectioned onto optimization slides, where it underwent permeabilization to capture the mRNAs, and was followed by generation of fluorescent cDNA. The intensity of the fluorescent signal from the epithelial layer was similar for within the span of 10–20 min permeabilization. Additionally, we noted that the hard structural dermal tissues, connective tissue, and bone required longer permeabilization times, compared with the epithelial tissue, to reach maximum intensity which was in the span of 25–35 min. After careful consideration of these findings, we decided to adopt a permeabilization time of 20 min for the tissue expression slides. This choice struck a balance between maintaining a satisfactory fluorescent signal for the soft epithelial tissues and achieving satisfying results for the harder dermal tissues. The relative long permeabilization time for the epithelial tissue increases the risk of RNA “diffusion” into neighboring capture areas and the subsequent loss of resolution associated with excessively long permeabilization times. Conversely, using a too short permeabilization time for the dermal tissue could result in low RNA yield. For laboratories which are planning to run the protocol, note that the optimal permeabilization time could vary depending on factors such as tissue condition (naïve vs disease) and the size and thickness of the tissues, which can be affected by the size and age of the animal.

**Fig. 3.**
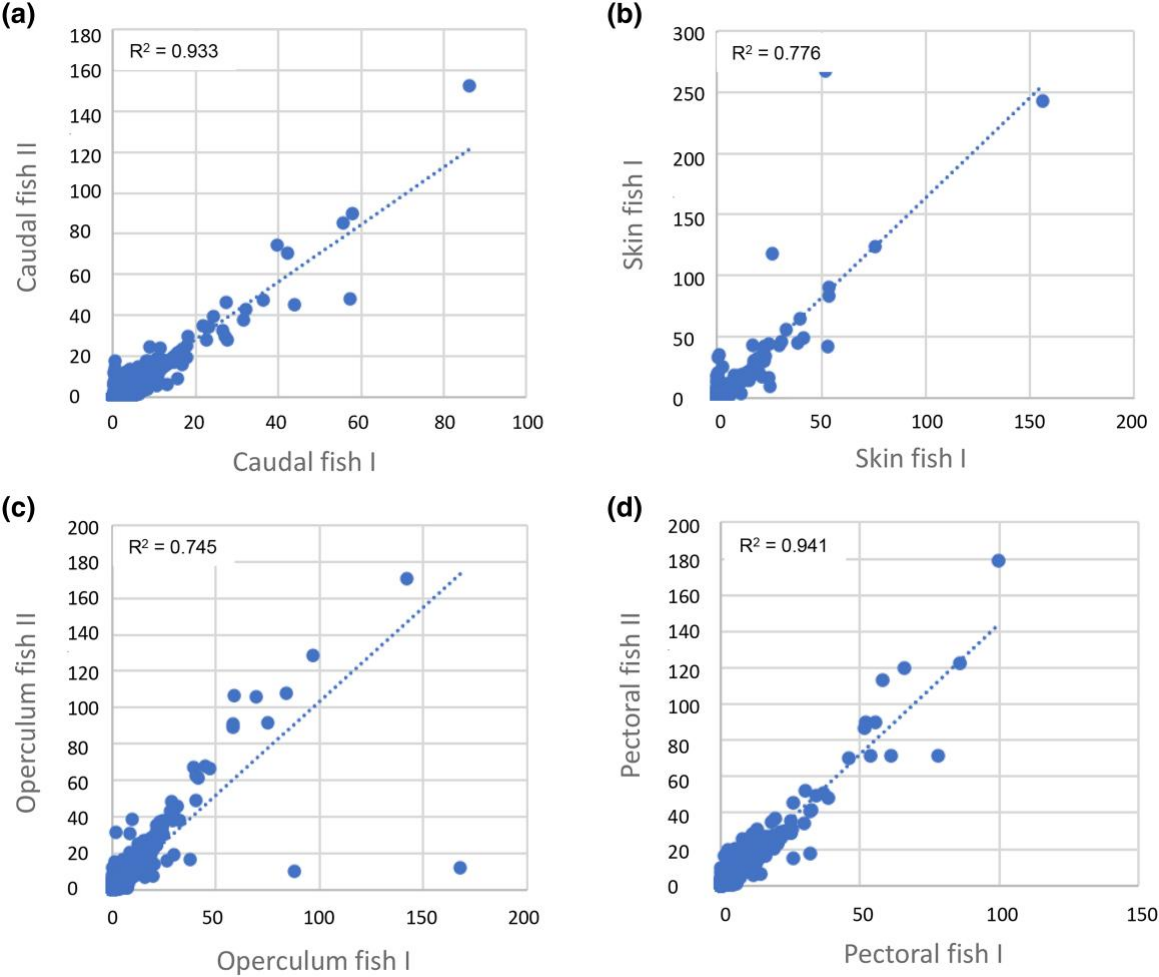
Normalized gene counts for Fish I and Fish II. Median-normalized average gene counts (X and Y axes) for Fish I and Fish II for skin tissue samples originating from the same position. a) Caudal fin, b) skin, c) operculum, and d) pectoral fin.

The number of reads obtained per sample varied between 81,982,019 and 103,270,656, with mapping efficiencies ranging from 79.3 to 89.4% (Supplementary File 1) indicating good library qualities. Good sample quality was further indicated by a linear correlation between normalized gene counts of Fish I and Fish II for similar samples (Fig. 3a–d) and further suggested a consistent gene expression profile across individuals.

Many transcripts were detected in all tissue sections, ranging from 29,292 distinct transcripts in the caudal fin of Fish II to over 33,000 in the caudal fin of Fish I (Supplementary File 1). Further, the average number of mapped transcript reads [median-normalization average (MNA)] per gene per spot was 0.148, and the median was only 0.006 (data not shown). Low transcript values are generated as spatial transcriptomics aims to count the number of transcripts of a gene at distinct spatial locations in a tissue; hence, it differs to that of bulk RNAseq analysis where gene counts are measured for an entire tissue sample. In Space Ranger, the MNA of a gene in a cluster is defined as the mean of observed UMI counts normalized by the size factor for each spot in the representative cluster. For this reason, genes that were expressed in multiple tissues (captured at multiple spots) had higher counts, compared with genes expressed by few cell types, or which were present only in 1 tissue.

### Epithelial tissue had the highest UMI counts

**Fig. 4.**
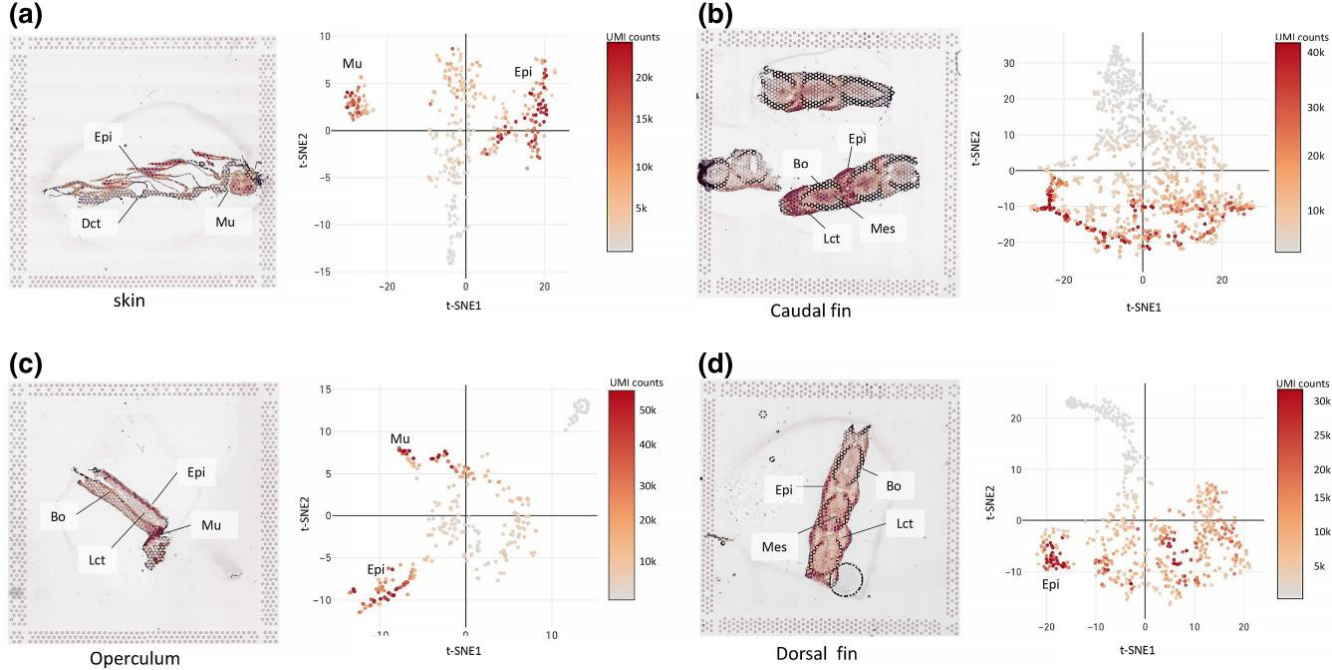
UMI counts in tissue from Fish I. a) Skin, b) caudal fin, c) operculum, and d) pectoral fin. For each sample, UMI counts are given as spots on top of the tissue section in the left panel, and the t-SNE projection with UMI counts is in the right panel. Epithelial tissue (Epi), muscle tissue (Mu), loose connective tissue (Lct), dense connective tissue (Dct), bone (Bo), and mesenchyme (Mes) are indicated in the plots.

The epithelial tissues exhibited the highest absolute number of observed transcripts (UMI counts) (Fig. 4), followed by muscle tissue, parts of the loose connective tissue in the caudal fin, and finally fibrous connective tissue and bone. These findings aligned with our earlier observations of fluorescent cDNA signal during sample preparation (Fig. 2d–f), where epithelial tissue had the strongest fluorescent signal, while the connective tissue compartments had a lower fluorescent intensity relative to the epithelial layer. This was as expected since densely populated tissues, such as the epithelial tissue, in general have a higher mRNA yield than sparsely populated tissues like connective tissue and bone.

### Assigning gene expression to spatial clustering of tissue types

**Fig. 5.**
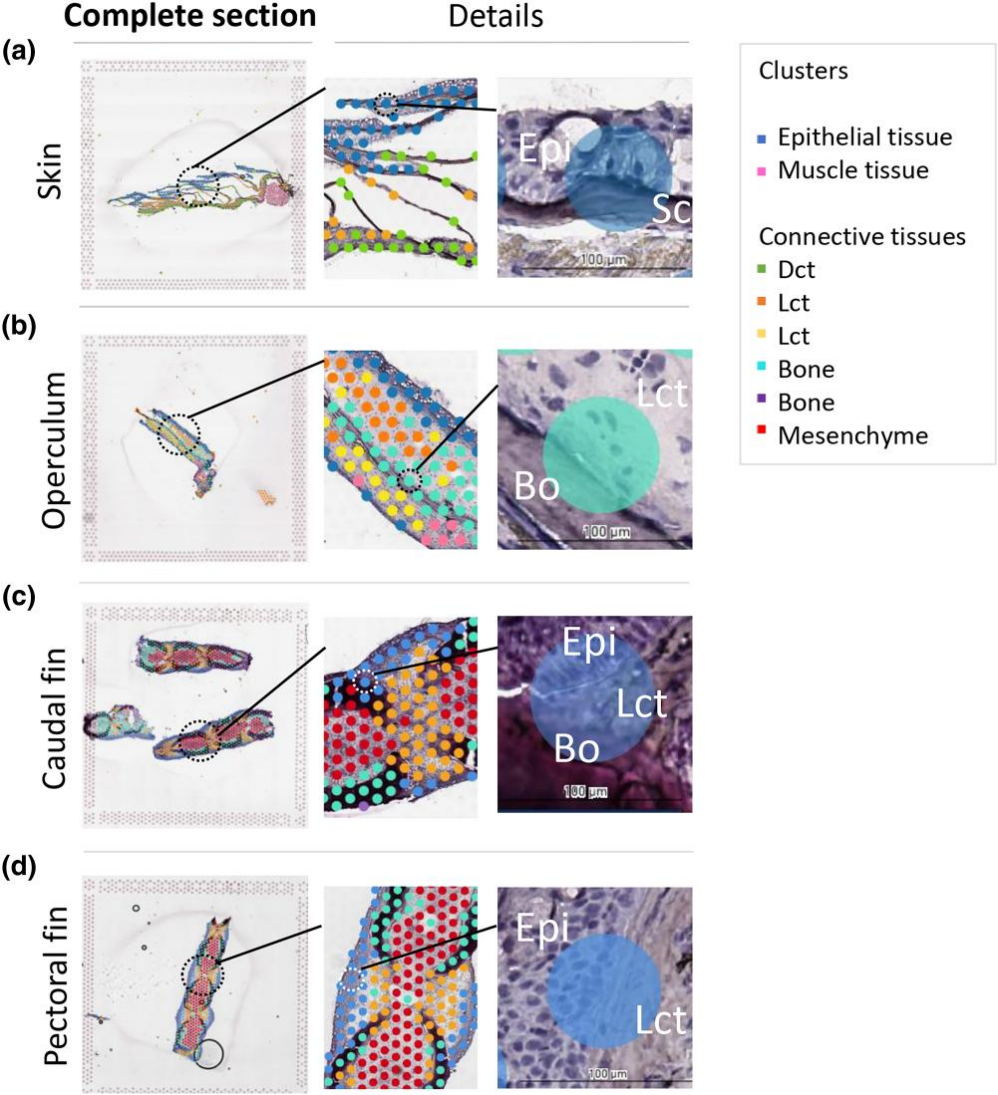
Illustrations of the graph-based clustering for Fish I. a) Skin, b) operculum, c) caudal fin, and d) pectoral fin. In each sample, the left picture displays the complete tissue section within 1 capture frame. Additionally, magnified areas are indicated by circles, showing detailed views of the complete section on the right side. The rightmost frame depicts a capture spot that encompasses multiple tissues. Clusters are represented by spots with similar colors, where each spot corresponds to a barcoded probe on the Visium expression slide. The tissue clusters were named based on the main tissue type present. Abbreviations: loose connective tissue (Lct), dense connective tissue (Dct), bone (Bo), and epithelium (Epi).

Within the spatial transcriptomics analysis workflow, assigning the gene expression in the capture spots to their spatial domains with unsupervised clustering is essential. We used Louvain graph-based clustering which gave 4–5 spatial clusters per sample (Supplementary File 1). The clusters were named according to the main tissue type present: epithelial tissue, loose and dense connective tissue, bone (fin ray and scales), mesenchyme, and muscle tissue (Fig. 5).

The graph-based clustering corresponded closely with our visual identification of tissue types (Fig. 5), although some clusters overlapped with more than 1 tissue. For instance, a cluster corresponding mainly with epithelial tissue overlapped with loose connective tissue and loose connective tissue with bone (Fig. 5d, pectoral fin), and in the operculum, the opercular bone also overlapped with loose connective tissue (Fig. 5b). In the skin, the scales were part of the epithelial cluster.

Since most genes are not cell or tissue specific, such as genes involved in core cellular functions like metabolism, DNA replication and repair, and protein synthesis, some overlap of transcriptomic profiles in the different tissues (and clusters) is expected. However, some overlap may also be due to the resolution of the Visium expression slides. Currently, the capture area on Visium expression slides is of 55 µm diameter with 100 µm center-to-center distance and 5,000 spots per array (Fig. 2). The presence of multiple tissue layers within a capture spot posed a challenge in obtaining clear clusters, as depicted in Fig. 5 (right panels). In such situations, mRNA from different tissue types combines in a single library. While overlapping regions primarily caused minor clustering errors in tissues like epithelial tissue, dense connective tissue, and mesenchyme, they presented a challenge for thinner tissues such as fish scales (Fig. 5a). Due to their size being smaller than the capture area, separate clusters for scales could not be formed.

It is further possible to fine-tune tissue clustering with other unsupervised methods such as increasing the number of clusters using k-means or with Seurat (Satija *et al*. 2015), stardust (Avesani *et al*. 2022), or GraphST (Long *et al*. 2023). Experimenting with cluster size, such as increasing k-means in the range of 6–10 clusters, resulted in improved arrangement of some of the clusters, such as more consistent overlay of the clusters for epithelial tissue in the pectoral fin sample (data not presented). However, increasing the cluster size also resulted in several smaller clusters which were not biologically meaningful. Hence, if the spatial libraries contain different cell populations, computational methods would not help without external data. Such external data could have been single-cell sequencing libraries (Baccin *et al*. 2020), which in combination with spatial transcriptomics would provide single-cell resolution while maintaining the positional information of expression.

### Transcriptional profiling of the skin

We further investigated the transcriptional profile of the tissue clusters. Using Fish I as an example, the avg. number of genes in a cluster was 235 (median 246). However, the number of genes assigned to a cluster varied between 8 DEGs for operculum and loose connective tissue and 507 DEGs for skin epithelial tissue. We further searched for unique genes within the clusters of a sample (Supplementary File 1). Here, unique means DEGs only being expressed in 1 cluster. For the skin tissues, the number of unique genes ranged from 18% in epithelial cluster of the pectoral fin to 0.5% in the epithelial tissue of operculum fin. Further, only 1 sample had muscle attached to the skin. The muscle tissue cluster had the highest number of unique genes (71%) compared with the epithelial and connective tissue of the skin in the same sample. In terms of validation of the technique, it is expected that muscle tissue, which is a different tissue type, and not present in the fins, stands out in terms of gene expression.

**Fig. 6.**
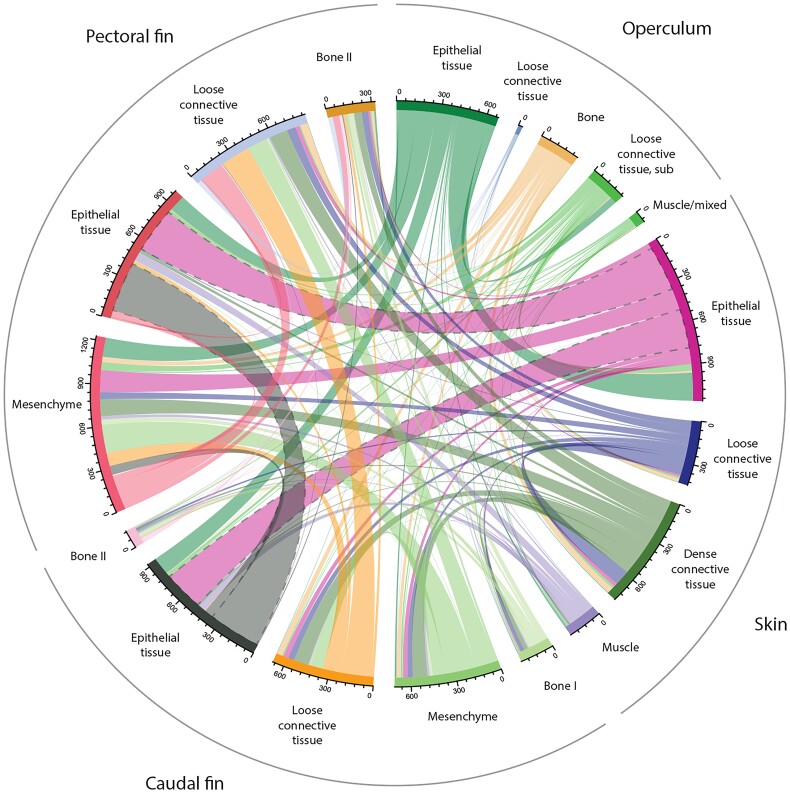
Chord diagram displaying the interrelationships between the transcriptional profiles within each cluster. The color and the thickness of the nodes visualize the relationships between the clusters. For operculum, “loose connective tissue, sub” refers to the loose connective tissue under the opercular bone.

Furthermore, the interrelationships among the transcriptomic profiles of tissue clusters revealed that epithelial clusters exhibited the broadest nodes (Fig. 6), indicating similarities in the transcriptome across different body sites. Conversely, multiple connections were observed between the dermal tissue clusters (Fig. 6), suggesting greater disparities in the transcriptome within dermal connective tissues at different body locations. It is important to note that in spatial transcriptomics, the composition of specific tissues or cell types present in a sample depends on the plane of the tissue section and the distribution of cell types within that plane. Therefore, given that epithelial tissue predominantly consists of keratinocytes and mucous cells (Eisenhoffer *et al*. 2017; Sveen *et al*. 2021b), a more comparable transcriptional pattern would be anticipated compared with dermal connective tissues, which encompass a diverse array of cell types (Whitear 1986a, 1986b) with distinct functions and gene expression patterns (Ferretti and Hadjantonakis 2019), in which structure and function varies with body site.

### *Collagen type 1*, the most abundant transcript in fish skin

Collagen is the most abundant structural protein in the extracellular matrix of the various connective tissues (i.e. skin, bones, ligaments, tendons, and cartilage), and fish skin is particularly rich in collagen (Jafari *et al*. 2020). Collagens provide structural support to ensure firmness, elasticity to the skin, and the strength that is needed for effective locomotion (Wainwright *et al*. 1978). At the protein level, fish skin typically contains collagen type 1 protein with a high degree of purity (around 70%), depending on the species age and season (Chinh *et al*. 2019), followed by collagen type 5 (Yata *et al*. 2001).

**Fig. 7.**
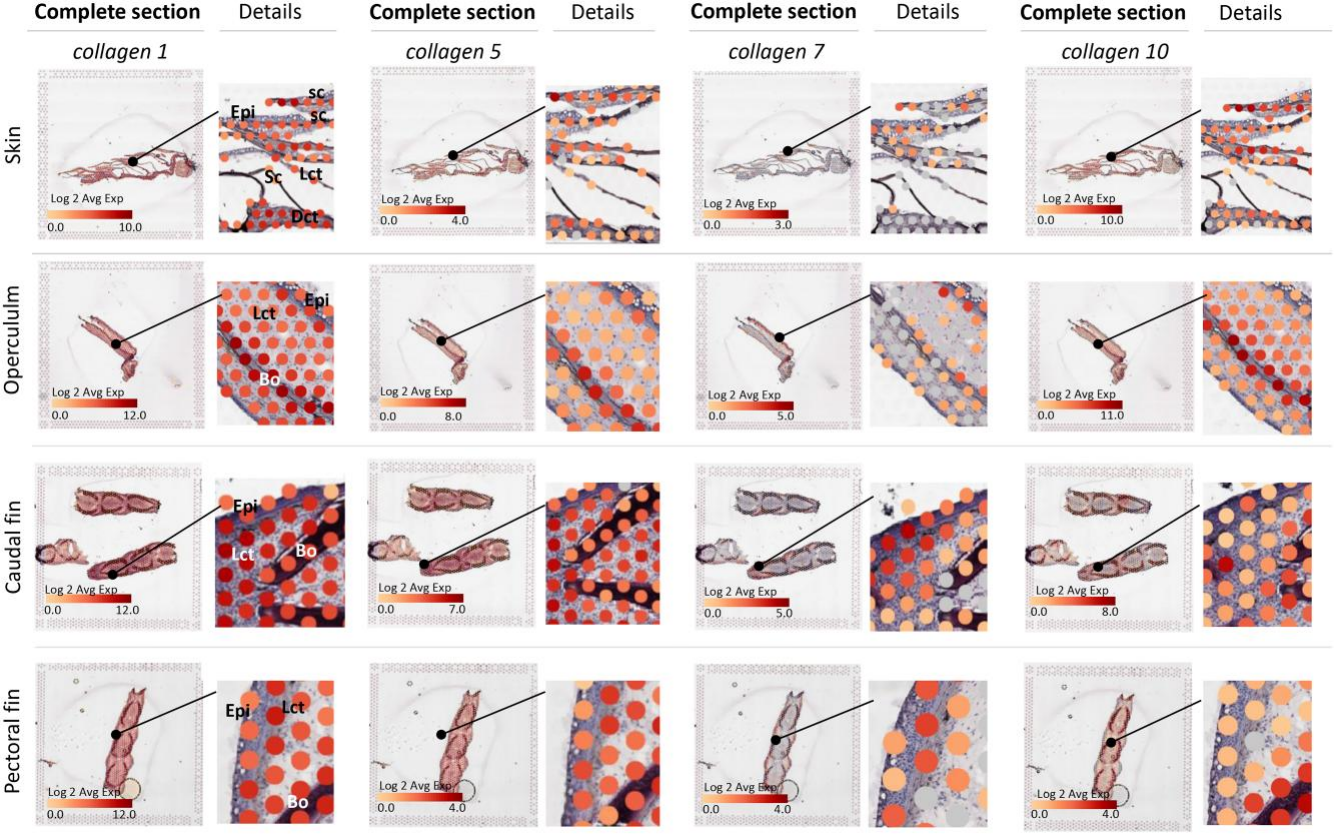
Expression of *collagen types 1*, *5*, *7*, and *10* in the skin, operculum, caudal fin, and pectoral fin. For each sample, the left picture shows the complete tissue section within the capture frame; magnified areas are indicated by black circles, and detailed views of the complete section are given on the right side. The figure illustrates the average gene expression for collagens annotated with the same names, as listed in Supplementary File 1, and the color of the capture spots indicate the Log2 expression. Abbreviations: epithelium (Epi), loose connective tissue (Lct), dense connective tissue (Dct), bone (Bo), scale (Sc), and mesenchyme (Mes). ENS ID of genes is given in Supplementary File 1.

In our data, 3 genes encoding *collagen type I* (*col1a1b*, *col1a2*, *col1a2)* were among the top 10 most highly expressed genes across all samples (Fig. 7; Supplementary File 1). These findings are consistent with previous discoveries by Micallef *et al*. (2012) and reflect the abundance of *collagen type 1* in fish skin. In our data, we further identified multiple genes encoding *collagen type 5* (*col5a1*, *col5a2b*, *col5a2a*, *col5a1*) (Fig. 7), which displayed a transcriptional pattern similar to collagen type I, albeit at lower levels. Collagen type 5 is a regulatory fibril-forming collagen (Mak *et al*. 2016) that plays a crucial role in the fibrillation of type 1 and 3 collagens (Seibel *et al*. 2006); thus, collagen type 5 is important for tissue quality.

While some genes annotated as *collagen type 5* exhibited comparable transcriptional responses, others showed variations in their spatial expression patterns. For instance, *col5a2b* had high specificity to bony features (Supplementary File 1), and it seems plausible that this gene is dedicated to fibril formation in bone, also in Atlantic salmon. In this regard, the spatial platform may also represent an initial or complementary tool for the investigation of neofunctionalization of duplicated genes in Atlantic salmon.

While encountering difficulties in clustering all transcripts into distinct tissue types, there were instances where the spatial resolution of individual genes provided promising results in accurately tracing them back to their respective tissue. This phenomenon can be illustrated through the examples of *collagen type 7* and *collagen type 10*. For example, *collagen type* 7 (*col7a1*) was expressed in the epidermal/dermal zone (Fig. 7), reflecting its role as a major component of anchoring fibrils that attach the epidermis to the dermis in vertebrate skin (Regauer *et al*. 1990). On the other hand, *collagen type 10* (*col10a1b*, *col10a1b*), which is in involved in the process of endochondral ossification in ray-finned fishes and tetrapods (Debiais-Thibaud *et al*. 2019), and specific marker for endochondral ossification in salmon (Ytteborg *et al*. 2010), was expressed near bony features, with almost perfect overlap with the opercular bone (Fig. 7).

### *Keratins* are abundant in fish epithelial tissue

Although keratins are perhaps best known for their role in cornified materials, they also play essential roles in differentiation and development of epithelial cells, cell growth/cycle, adhesion, and stress response (Moll *et al*. 2008; Bragulla and Homberger 2009). Keratin proteins are interfilamentous proteins which extend from the cell nucleus to the plasma membrane, attach to desmosomes, and interact with a variety of cell structures, thereby contributing to the tensile strength and shape of the cell, likely aiding in withstanding mechanical stress (Schaffeld and Markl 2004).

**Fig. 8.**
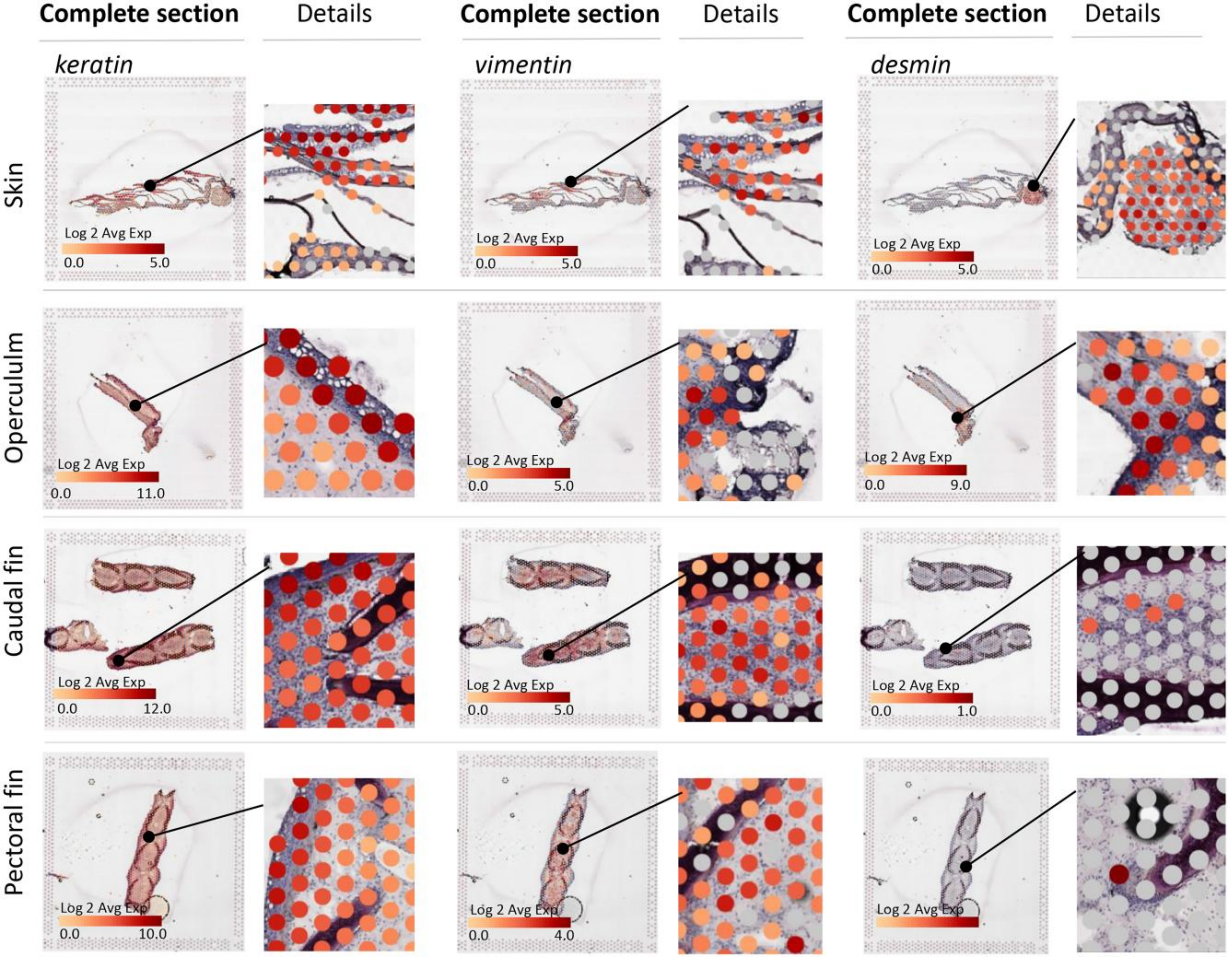
Expression of interfilamentous proteins in the skin, operculum, caudal fin and pectoral fin. For each sample, the left picture shows the complete tissue section within the capture frame; magnified areas are indicated by black circles, and detailed views of the complete section are given to the right. *Keratins* had the overall highest expression rates in the fin and operculum. In the skin, they were primarily expressed in the epithelial layer. *Vimentin* (ENS0000073113) was expressed around scale pockets in the skin, in a fold in the operculum, and primarily in the mesenchyme of the fins. *Desmin* (ENS00000101128 and ENS0000040563) was transcribed in skeletal muscle tissue attached to the skin sample and in the opercular fold, with limited expression in fins. The figure displays keratin genes as the average gene expression of “keratins” (as listed in Supplementary File 1), and the color of the spots indicates Log2 expression.

In Atlantic salmon skin epithelia, several *keratins* (*krt15*, *krt5*, *krt8*, *krt18*) were highly expressed (Fig. 8; Supplementary File 1). The fact that these *keratins* were differentially expressed in epithelial tissue during naive conditions implies that they are important for normal growth and maintenance of the epithelium. In mammalian cells, keratins also play a role in the keratinocyte activation cycle (Freedberg *et al*. 2001), where the keratinocytes turn into migratory and hyperproliferative cells. Several keratins, *keratin8* (Schaffeld and Markl 2004) and *keratin15* (Murawala *et al*. 2017), are also expressed at high level in the epidermis of regenerating caudal fin, illustrating that keratins are of particular importance during skin repair and regeneration.

Furthermore, it is worth noting that keratin proteins are frequently expressed in pairs, with each pair being reliant on one another for proper filament assembly (Ho *et al*. 2022). In our data set, we observed the presence of the *keratin8* and *keratin1*8 pair. This keratin pair is shared among all vertebrates (Kimura and Nikaido 2021) and resembles most closely the ancestral precursor of all other keratins (Krushna Padhi *et al*. 2006), In mammals, *keratin5* typically pairs with *keratin14*, while *keratin15* does not require pairing and serves as a marker for epidermal stem cells, often coexpressed with *keratin5*/*keratin14* (Bose *et al*. 2013). Notably, there are currently no genes annotated as *keratin14* in the salmon genome (Ssal_v.3.1). When comparing genes across different species, especially those with diverse evolutionary histories, determining which gene in 1 species corresponds to a gene in another species becomes a challenge. Moreover, the expression specialization or pairing of interfilament proteins is not always straightforward; keratin proteins may become dispensable in some species and repurposed in other assemblies (Ho *et al*. 2022). Therefore, gaining a comprehensive understanding of the keratins in *A. salmon* skin would require a more targeted and focused analysis.

Further, in terrestrial animals, keratin expression is mostly restricted to epithelial cells. In lower vertebrates, however, immunoreactivity for *keratin8* and *18* has been reported in nonepithelial cells and in mesenchymal progenitor cells of regenerating limbs in urodele amphibians (Corcoran and Ferretti 1997). In teleost fish, mesenchymal cells also express keratins (Groff *et al*. 1997; Conrad *et al*. 1998); hence, this might explain why we observe keratin expression in multiple tissue clusters, particularly in the fin (Fig. 8). Further, in non-teleost vertebrates, mesenchymal-derived cells usually do not express keratins but another type of interfilamentous protein termed vimentin (Herrmann *et al*. 1989; Schaffeld and Markl 2004). In zebrafish, vimentin has a key function in fin regeneration, working downstream of wound-induced redox signaling where it regulates collagen expression and reorganization (LeBert *et al*. 2018). Vimentin in turn is structurally closely related to desmin, another interfilamentous protein expressed in muscle cells (Schaffeld *et al*. 2001; Kürekçi *et al*. 2021). In our data, *vimentin* expression partly resembled that of keratins, with expression in the epithelial layer of the skin and mesenchyme of the fins (Fig. 8). Conversely, *desmin* displayed high expression in skeletal muscle tissue, as well as in the operculum near the levator opercula muscle, along with other muscle-associated genes. These findings demonstrate the potential of spatial transcriptomics in verifying the spatial expression patterns of keratins, *vimentin*, and *desmin*, in which expression patterns across different tissues are well established.

The high keratin content of fish epithelial tissue has been recognized for decades, and the epithelial cells were early on named filament-containing cells (Henrikson and Gedeon Matoltsy 1967), and later literature have referred to fish epithelial cells keratinocytes (Lee *et al*. 2014) and keratocytes (Lee *et al*. 1993). Among the abovementioned terms, “keratocytes” and “keratinocytes” are most used to describe the fish epithelial cells. However, “keratocyte” can be misleading as it also refers to mesenchymal cells in the corneal stroma, which have distinct functions and fate (West-Mays and Dwivedi 2006). Although “keratinocytes” accurately describe the high expression of keratin in fish epithelial cells, it does not provide a distinctive name that sets them apart from their mammalian counterparts. Therefore, the proper choice of terminology for fish skin epithelial cells remains a matter of consideration and would benefit from scientific discourse.

### Manual curation for tissue-specific gene markers

**Fig. 9.**
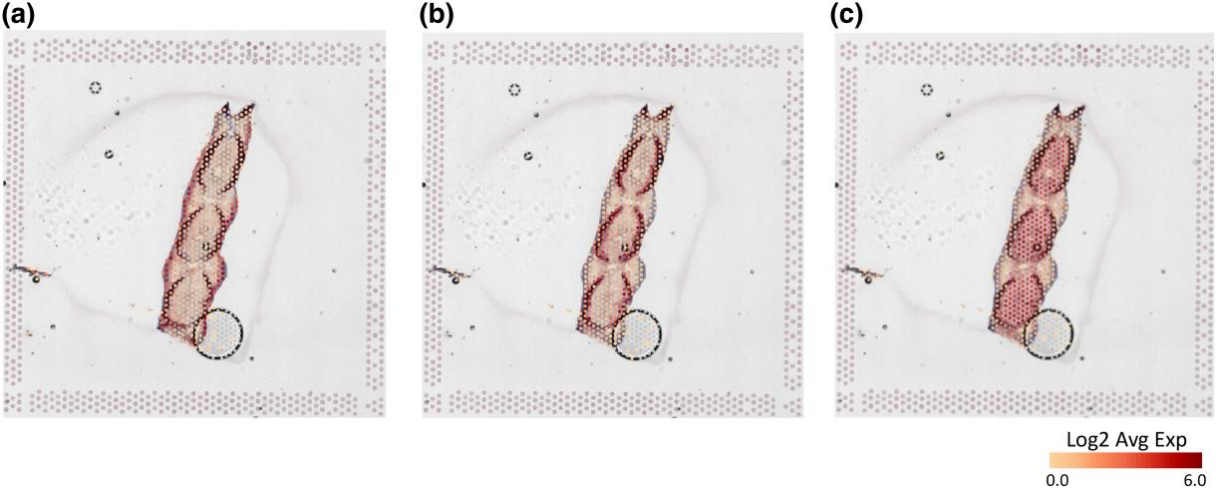
Expression of marker genes in the epithelium, bone, and mesenchyme. a) Pectoral fin, epithelial gene markers; b) pectoral fin, bone gene markers; and c) pectoral fin, mesenchymal markers. For each tissue section, the AVG Log2 expression is marked by colored spots on top of the tissue section. The marker genes behind the avg. expression ratio are listed in Supplementary File 1.

As different types of tissues have unique gene expression profiles and certain genes may be specifically expressed in certain tissue types, gene markers are useful for identifying specific tissues. We manually searched through DEGs within tissue clusters and looked for DEGs within epithelial tissue, bone, or mesenchymal tissue to identify gene markers. This resulted in a list of genes in which the transcription primarily corresponded to 1 tissue (Fig. 9; Supplementary File 1). We noted that the epithelial gene markers were more consistently expressed within the epithelial clusters than the suggested gene markers for the bone and connective tissue. The difficulties of finding gene markers of connective tissues have been exemplified in other experiments and are partly due to the embryonic origin of the cell types and the ability of mesenchymal cells to transdifferentiate into other cell types (Ytteborg *et al*. 2015).

**Fig. 10.**
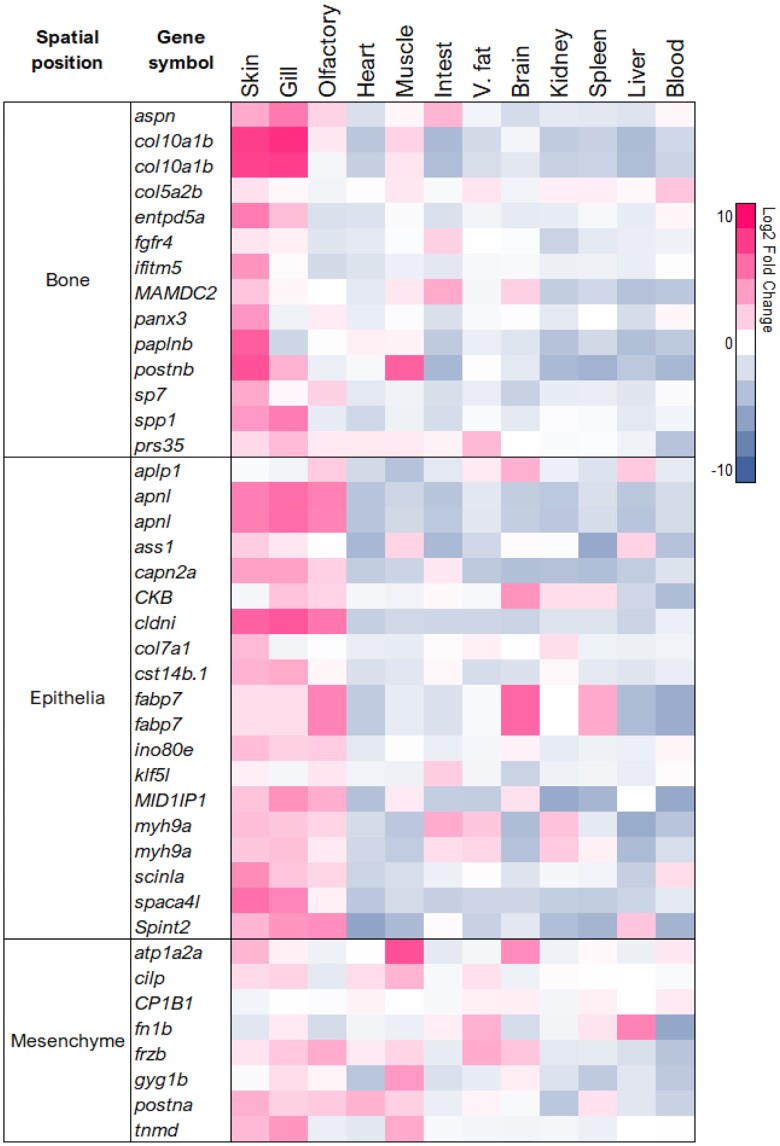
Distribution of gene markers and their transcripts in the major tissues and organs of Atlantic salmon. Data are Log2 AVG fold change of tissue to the mean of all tissues according to Nofima’s microarray database STARS (Krasnov *et al*. 2011). ENS ID of genes is given in Supplementary File 1.

To validate the identified gene markers, we checked the distribution of their transcripts in the major tissues and organs of Atlantic salmon. These data were available in Nofima's bioinformatic system STARS (Krasnov *et al*. 2011) that stores a large volume of transcriptome data obtained with 44k Atlantic salmon genome-wide microarray. As expected, the gene markers exhibited high expression in the skin (Fig. 10), showing notable similarities with the other key mucosal organs such as the gills and olfactory rosette, 2 organs that share immunological features (Lazado *et al*. 2023). The intestine, also categorized as a mucosal organ, demonstrated lower similarity to the skin than the gill and olfactory rosette, with most gene markers showing lower expression. The skin, gill, and olfactory rosette share greater structural similarity due to their combination of soft and hard tissues, in contrast to the intestine, which is predominantly composed of soft tissues. This difference in tissue composition could potentially account for variations in expression profiles across the mucosal tissues. Further, the brain, kidney, spleen, liver, and blood displayed overall low expression of the gene markers, although a few exceptions were observed, such as the high expression of *fatty acid binding protein 7* in the brain and spleen. In terms of validating the spatial technology for new species, it was encouraging to find concordance of results produced with different methods.

Research into vertebrate bone development has been extensively explored (Dietrich *et al*. 2021), and the bone markers identified in this study have previously been associated with bone development. Notable examples include secreted phosphoprotein 1 (spp1), also called osteopontin (Fonseca *et al*. 2007), asporin (aspn) (Lorenzo *et al*. 2001), and interferon-induced transmembrane protein 5 (ifitm5) (Moffatt *et al*. 2008). These genes are important for bone mineralization, and furthermore, osteopontin (spp) and periostin (postn) hold significant roles in bone remodeling and repair, interacting with extracellular matrix proteins to influence bone formation and integrity (Noda and Denhardt 2008; Gorski 2011). Furthermore, key genes for skeletal development are fibroblast growth factor receptor 4 (fgfr4) (Gebuijs *et al*. 2022) and sp7 (osterix), which is involved in fin regeneration (Dietrich *et al*. 2021).

In the context of salmon aquaculture, health issues related to skeletal disorders are concerning both during early development (Robinson *et al.* 2021), during production as excessive stress factors such as crowding can delay wound healing and scale mineralization (Sveen *et al*. 2018), and at the slaughter line (Holm *et al*. 2020). As such, these gene markers could prove valuable for future research concerning skeletal development in Atlantic salmon.

**Fig. 11.**
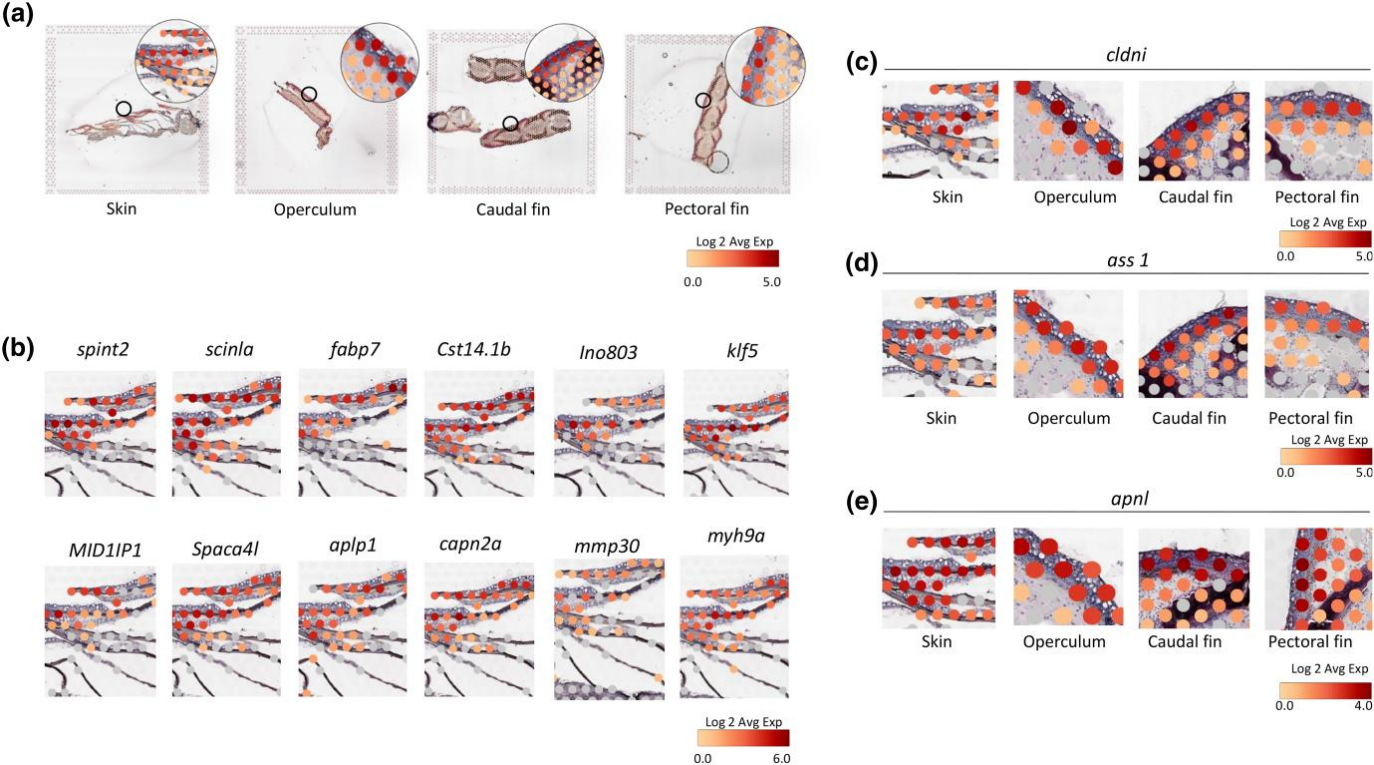
Expression of epithelial gene markers in the skin. a) Avg. Log2 expression of all epithelial gene markers listed in Supplementary File 1, for the skin, operculum, caudal fin, and pectoral fin. Magnified areas of each section are in the upper right corner, and area of magnification is marked by circles on the main slide. b) Expression of epithelial gene markers in skin epithelium. c and d) Expression of *cldni*, *ass1*, and *apnl* in the epithelium of the skin, operculum, caudal fin, and pectoral fin. Note that the complete slides with AVG expression ratios are depicted in a, while b–e only display magnified areas of the original slide, and each picture represents 1 marker gene.

For the identified epithelial gene markers, a few have well-annotated functions, such as *claudin I* (*cldni*) (Fig. 11), which belongs to a family of tight junction proteins and plays an important role in maintaining tight junctions between adjacent epithelial cells, preventing the leakage of solutes across the tissue (Doyle *et al*. 2022). Another gene which is well characterized in fish skin epithelial cells are the *myosins* (*myh9a*) (Okimura *et al*. 2018). Myosins constitute a large family of contractile proteins (Lazado *et al*. 2014). Epithelial myosins are motor proteins, which together with actin (microfilaments) are the major proteins involved in migration of the epithelial cells (Okimura *et al*. 2018), which is particularly important during development and wound healing (Richardson *et al*. 2016). In our previous work, we have encountered myofiber transcripts in fish skin as a response to salmon lice (Skugor *et al*. 2008; Tadiso *et al*. 2011; Krasnov *et al*. 2012), chemotherapeutic treatment (Lazado *et al*. 2021), and wound healing (Sveen *et al*. 2019), and this suggests the importance of epithelial cell migration not only with skin damage but also with parasitic infection and other hazardous treatments. Further, *kruppel-like factor 5-like* (*klf5l*) belongs to a family of transcription factor that plays a role in cell proliferation and differentiation (McConnell and Yang 2010). In salmon skin, *kruppel-like factors* are differentially expressed in damaged tissues (Sveen *et al*. 2019), with lice (Holm *et al*. 2017), and chemical treatment (H_2_O_2_ exposure) (Karlsen *et al*. 2012). Considering existing research, the identification of these epithelial markers underscores the active engagement of skin epithelium in promoting skin resilience. However, while certain roles of the identified epithelial gene markers have been clarified, many others remain incompletely understood, demanding further investigation.

## Concluding remarks

Overall, the findings presented in this study highlight the potential for achieving high spatial resolution of skin tissues in Atlantic salmon. While the overarching task of accurately classifying all transcripts into distinct tissue clusters remains challenging, the ability to trace the spatial localization of specific genes with precision opens new avenues for understanding tissue-specific gene expression patterns. It is important to note that while these individual gene examples showcase promising results, comprehensive analysis necessitates a broader examination and integration of multiple genes within the context of tissue morphology. Nevertheless, the ability to accurately trace the spatial resolution of single genes to their spatial origin signifies a significant step forward in unraveling the intricate dynamics of gene expression within complex biological systems.

As the field of spatial transcriptomics continues to advance, we expect this technique to become an indispensable tool for comprehensive molecular characterization and mapping of diverse tissues and diseases in Atlantic salmon. The integration of spatial transcriptomics with other omics technologies, such as single-cell RNA sequencing and spatial proteomics, will further enhance our understanding of complex biological systems. Looking ahead, our future work will involve the integration of the aforementioned omics techniques. Specifically, we will focus on comparing naïve skin tissue with diseased samples, including skin ulcers, and investigating the attachment site of parasitic salmon lice. These endeavors aim to capture molecular markers associated with wound repair processes or host susceptibility. Ultimately, the insights gained from spatial transcriptomics will drive advancements in fish health management, disease prevention, and therapeutic interventions in aquaculture settings.

## Supplementary Material

jkad215_Supplementary_Data

## Data Availability

All sequence data have been submitted to the Sequence Read Archive (SRA) as BioProject PRJNA970983. JPG files of tissue sections are given in Supplementary File 2. Supplemental material available at G3 online.
