# Supplementary material for: Transcriptomic landscape of Atlantic salmon (Salmo salar L.) skin
Source: G3 (Bethesda). 2023 Sep 19;13(11):jkad215. doi: 10.1093/g3journal/jkad215 (PMC10627282; doi:10.1093/g3journal/jkad215)
Supplement: jkad215_Supplementary_Data [file jkad215_supplementary_data.zip › File_S2_G3-2023-404525.pptx]

## Slide 1
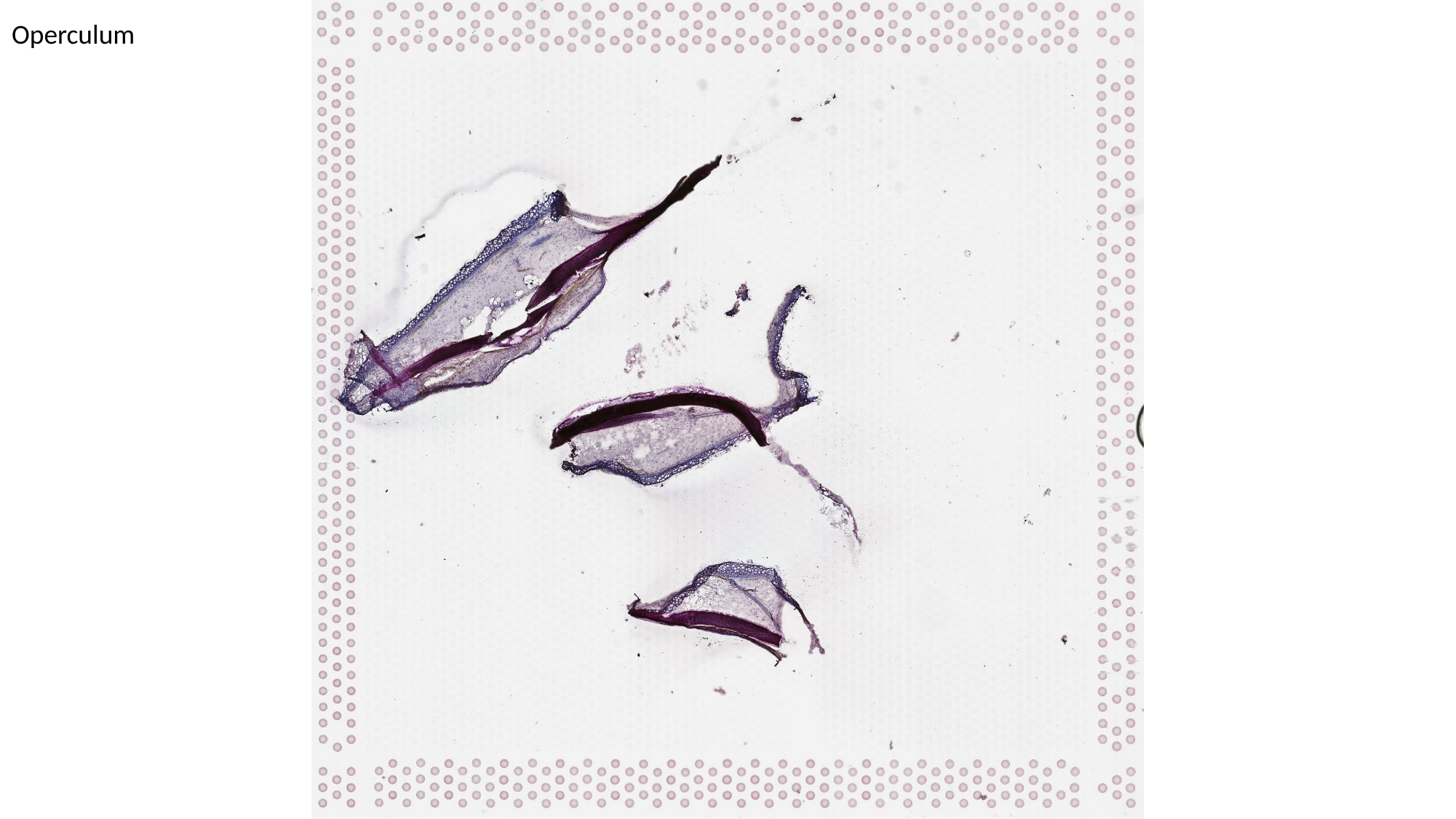

Operculum

## Slide 2
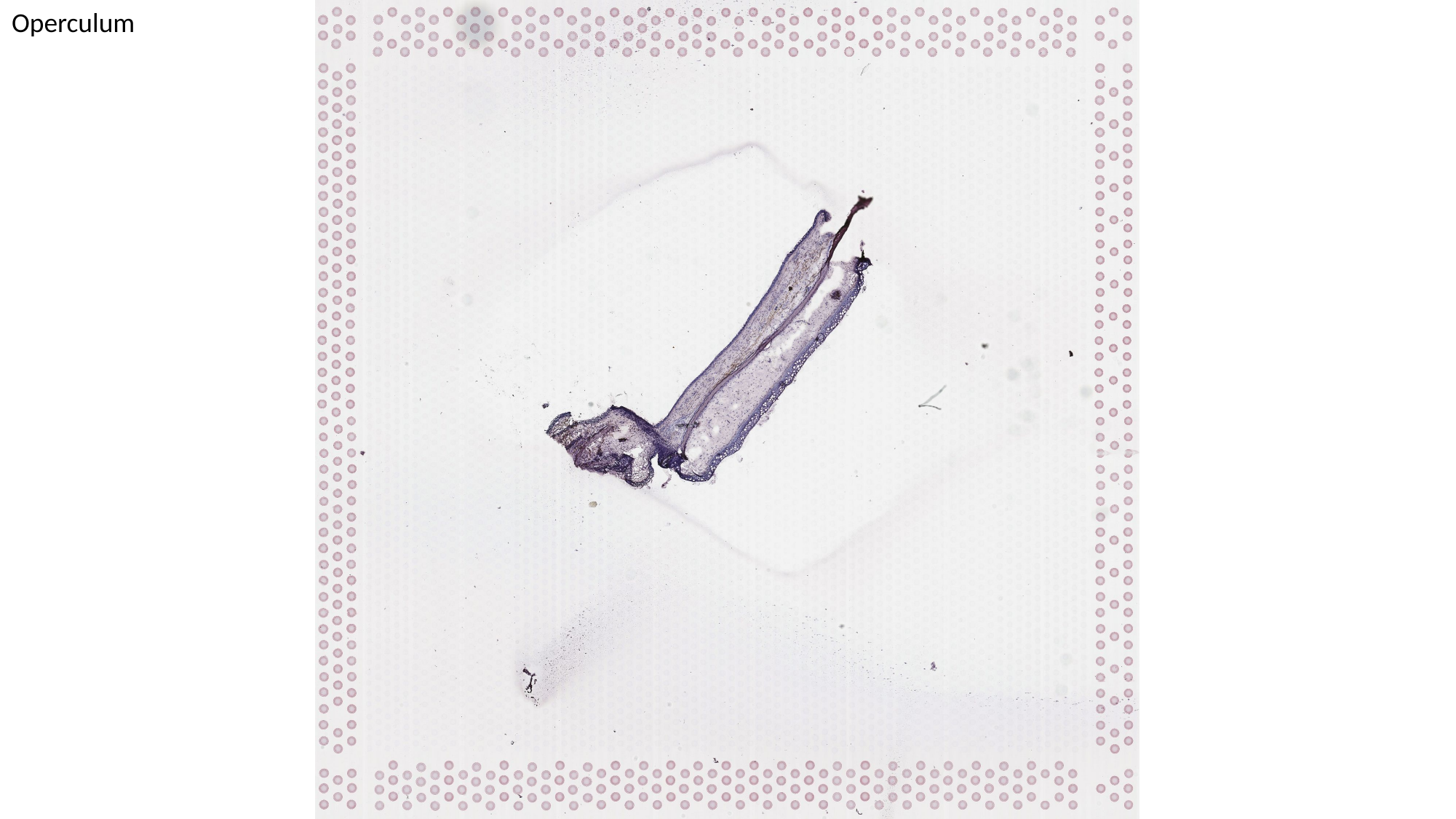

Operculum

## Slide 3
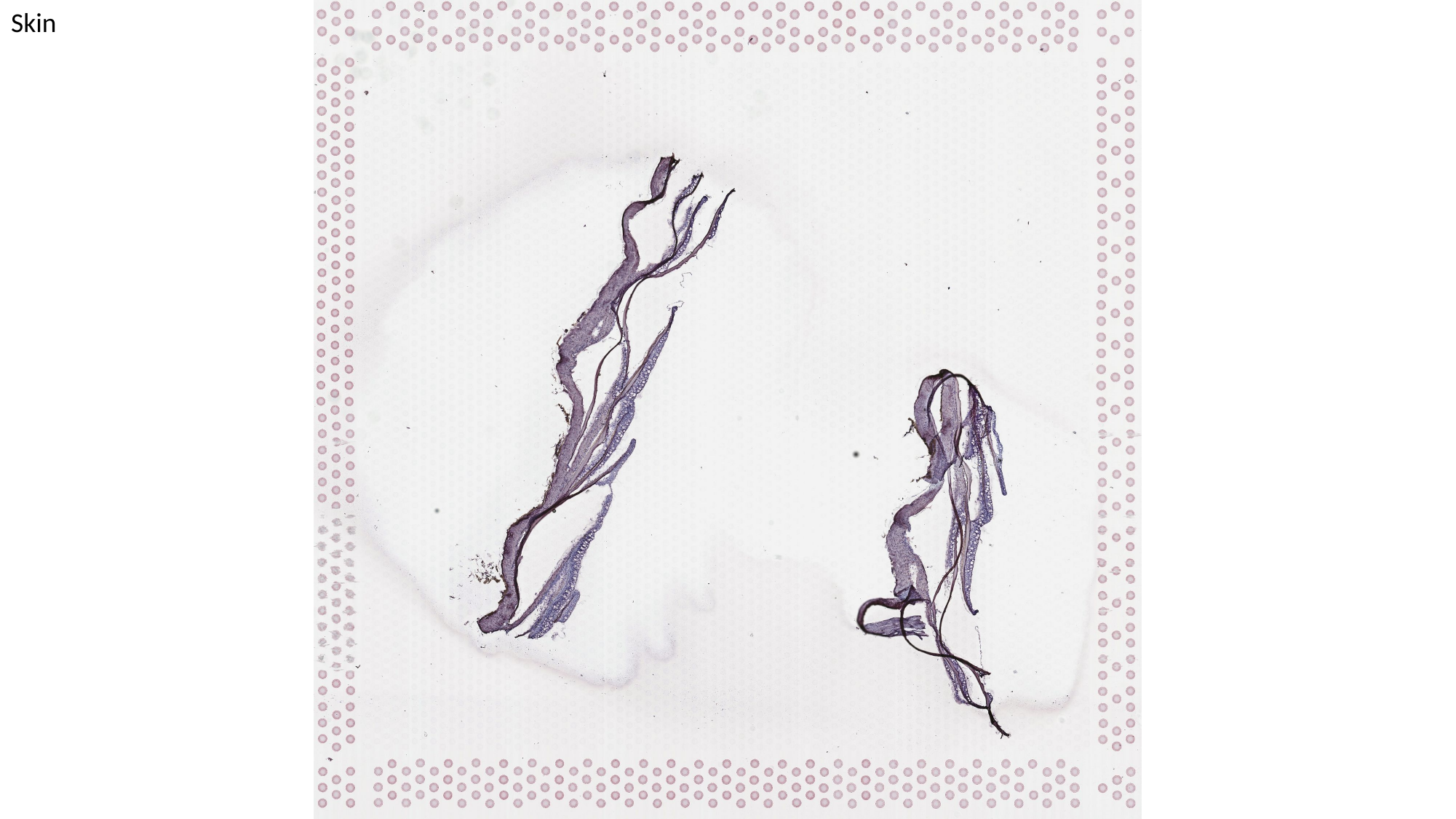

Skin

## Slide 4
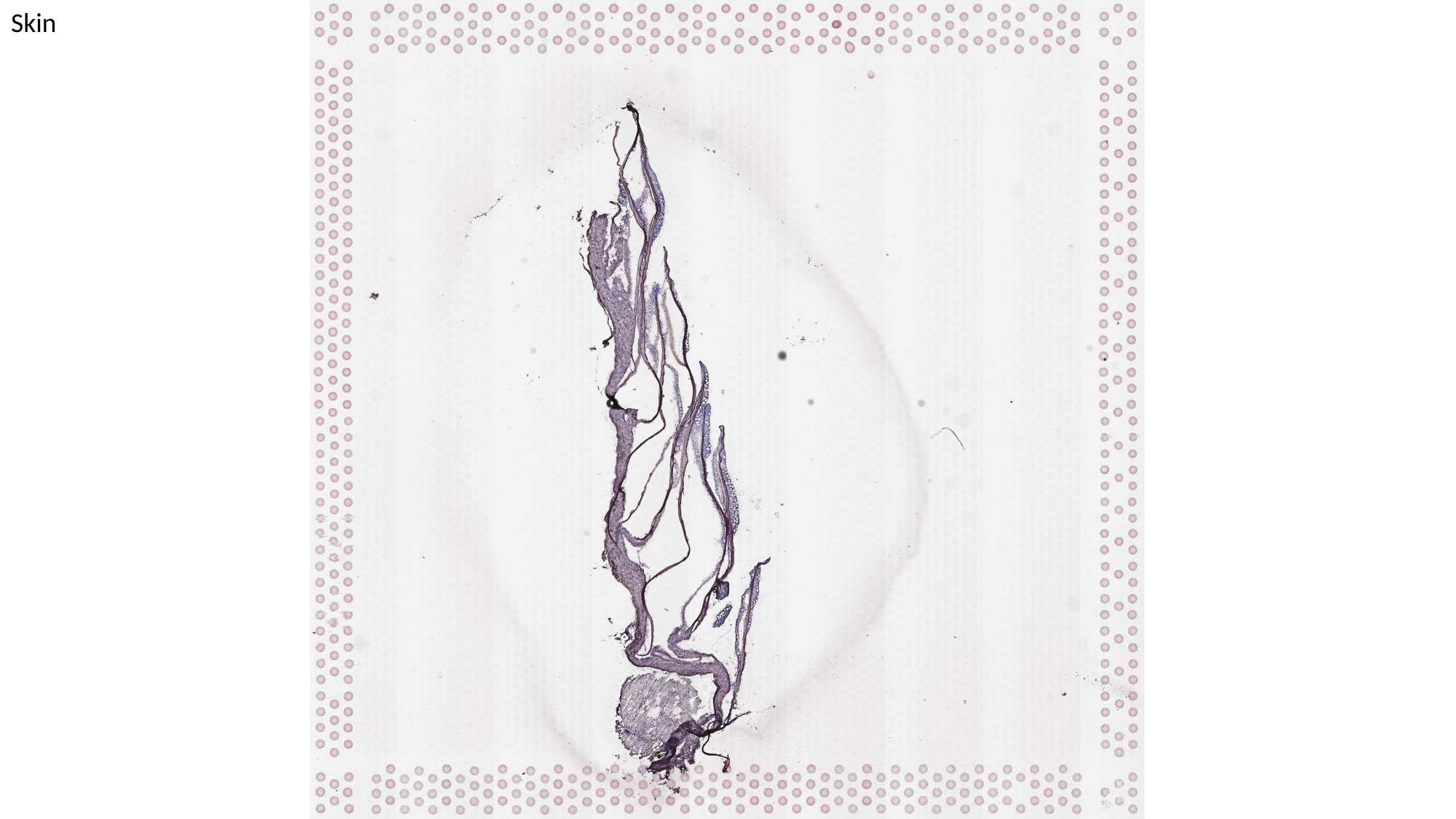

Skin

## Slide 5
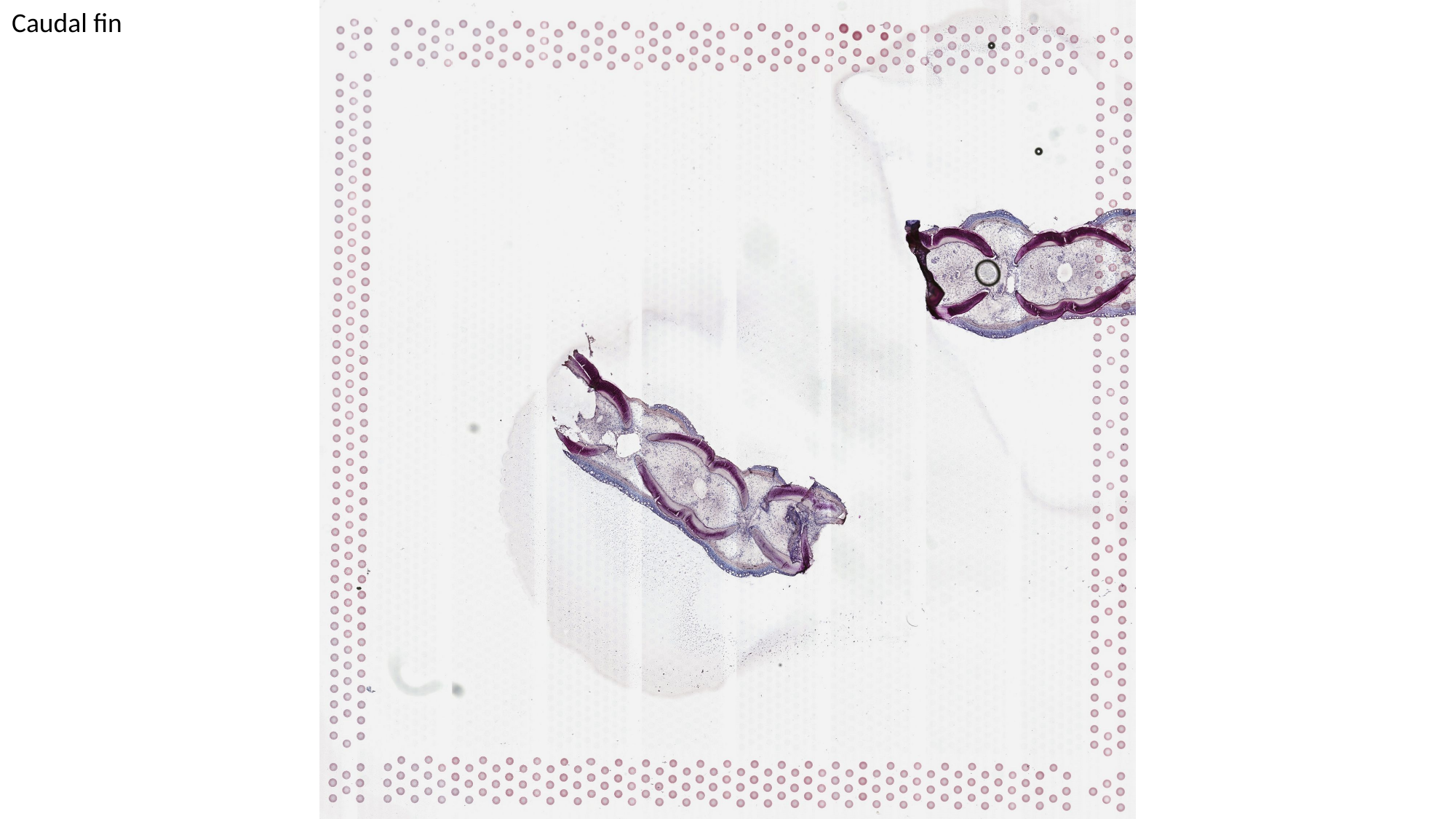

Caudal fin

## Slide 6
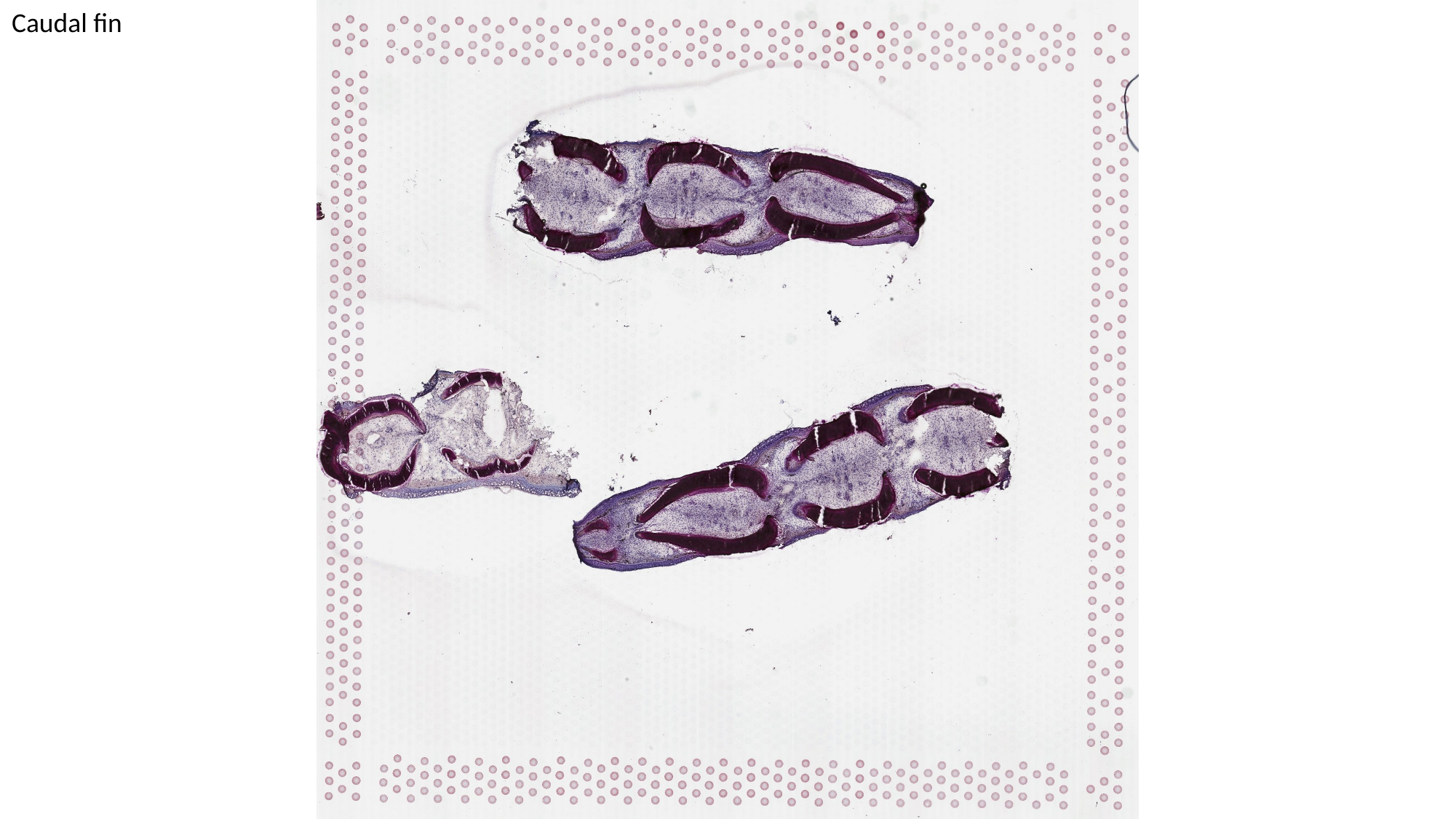

Caudal fin

## Slide 7
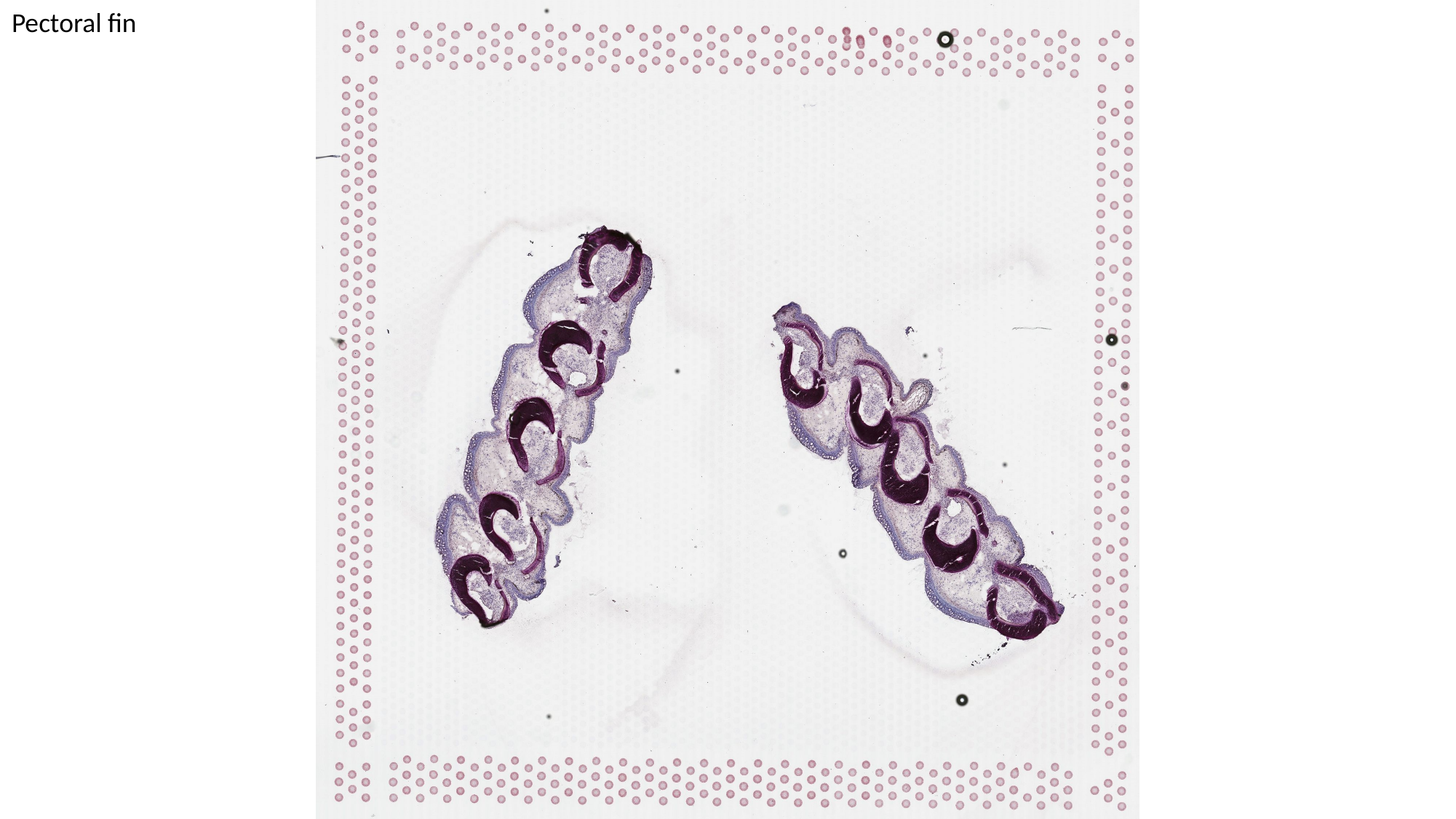

Pectoral fin

## Slide 8
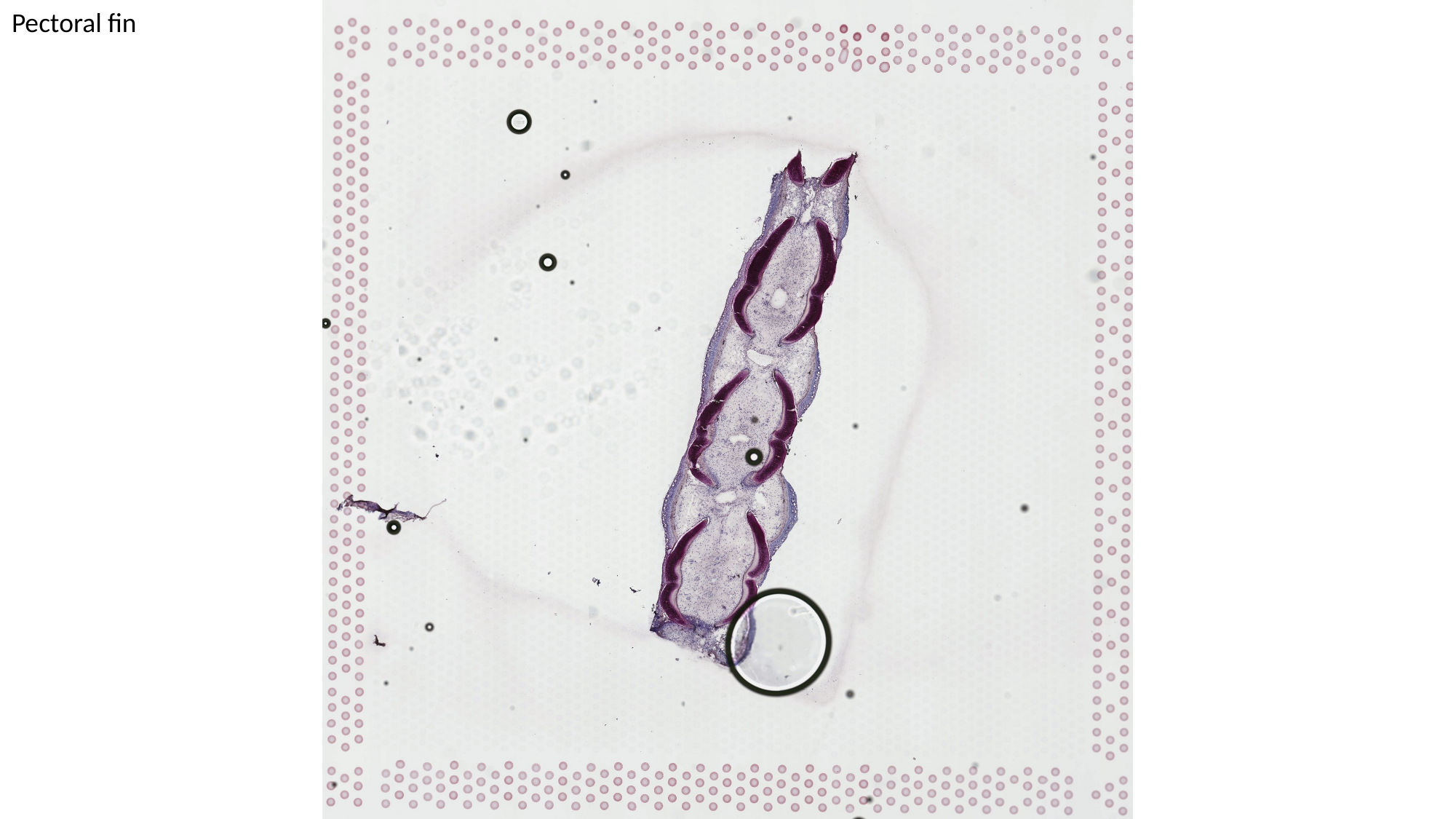

Pectoral fin
